# Study of infectious diseases in archaeological bone material – A dataset

**DOI:** 10.1016/j.dib.2017.06.054

**Published:** 2017-07-04

**Authors:** Elisa Pucu, Paula Cascardo, Marcia Chame, Gisele Felice, Niéde Guidon, Maria Cleonice Vergne, Guadalupe Campos, José Roberto Machado-Silva, Daniela Leles

**Affiliations:** a Laboratório de Biologia Molecular de Parasitos e de Paleoparasitologia, Departamento de Microbiologia e Parasitologia, Instituto Biomédico, Universidade Federal Fluminense, Rua Professor Hernani Melo, n. 101, Bairro São Domingos, Niterói, Rio de Janeiro, Brazil; b Laboratório de Paleoparasitologia, Escola Nacional de Saúde Pública, Fiocruz, Rio de Janeiro, Rio de Janeiro, Brazil; c Fundação Museu do Homem Americano, São Raimundo Nonato, Piauí, Brazil; d Universidade Federal do Vale do São Francisco, Campus Serra de Capivara, São Raimundo Nonato, Piauí, Brazil; e Universidade do Estado da Bahia, Salvador, Brazil; f Museu de Astronomia e Ciências Afins, Rio de Janeiro, Rio de Janeiro, Brazil; g Universidade do Estado do Rio de Janeiro, Brazil

**Keywords:** Paleoparasitology, Paleomicrobiology, ancient DNA, Taphonomy

## Abstract

Bones of human and ground sloth remains were analyzed for presence of *Trypanosoma cruzi* by conventional PCR using primers TC, TC1 and TC2. Sequence results amplified a fragment with the same product size as the primers (300 and 350pb). Amplified PCR product was sequenced and analyzed on GenBank, using Blast. Although these sequences did not match with these parasites they showed high amplification with species of bacteria. This article presents the methodology used and the alignment of the sequences. The display of this dataset will allow further analysis of our results and discussion presented in the manuscript “Finding the unexpected: a critical view on molecular diagnosis of infectious diseases in archaeological samples” (Pucu et al. 2017) [1].

**Specifications Table**

**Table:** 

Subject area	Biology
More specific subject area	Molecular Biology and Paleoparasitology
Type of data	Figure, Table and Text File
How data was acquired	Automatic Sequencer (Applied Biosystems); Chromas Lite 2.1; BioEdit 7.2.5
Data format	Analyzed
Experimental factors	DNA extracted from ancient bone samples
Experimental features	We extracted DNA from Bone samples and tested for *Trypanosoma cruzi* with conventional PCR
Data source location	Justino Site, Sergipe, Brazil; Funerary Site São Gonçalo Garcia Church, Rio de Janeiro, Brazil; Lagoa dos Porcos Site, Piauí, Brazil. Data was analyzed at Fluminense Federal University, Niterói, Brazil.
Data accessibility	The data are available with this article

**Value of the data**

• Data will be useful to investigate differences in DNA sequence results
• This data allows the comparison with samples of similar archeological context.
• Data can be used to investigate methodologies and primers’ design.

## 1 Data

**Fig. 1:**
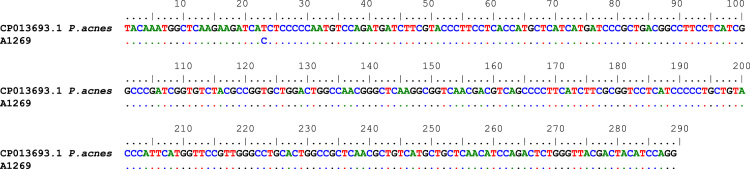
Sequence alignment of archeological sample A1269 obtained with the pair of primers TC/TC1 compared to the fragment of *Propionibacterium acnes* (Genbank access# CP013693.1) with 99% of similarity (Symbols:.=similarity).

**Fig. 2:**
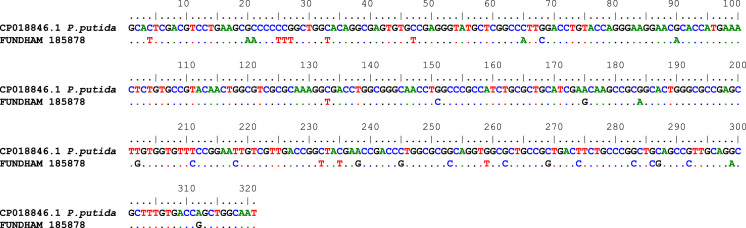
Sequence alignment of archeological sample FUNDHAM 185878 obtained with the pair of primers TC/TC1 compared to the fragment of *Pseudomonas putida* (Genbank access# CP018846.1) with 89% of similarity (Symbols:.=similarity).

**Fig. 3:**
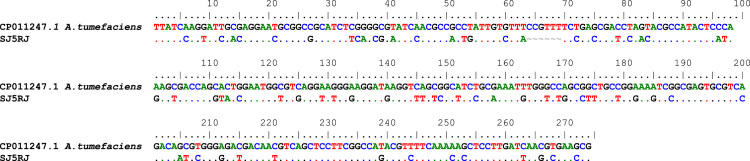
Sequence alignment of archeological sample SJ5RJ obtained with the pair of primers TC/TC1 compared to the fragment of *Agrobacterium tumefaciens* (Genbank access # CP011247.1) with 71% of similarity (Symbols:.=similarity; ~=gaps).

We present data from bones of human remains from Brazil: Justino site, Sergipe (*n*=7), dated from 4380–3200 BP (Before Present); and Funerary Site São Gonçalo Garcia Church, Rio de Janeiro (*n*=7) dated from the end of the 18th century [1]. Some (*n*=5) bone fragments were also analyzed from an individual extinct giant ground sloth of the genus *Eremotherium* spp., from Lagoa dos Porcos site, Piauí, Brazil dated from 30.000 BC (Before Christ) [1]. Data include methodologies, primers’ information and conditions (Table 1) and sequence alignment. (Fig. 1, Fig. 2, Fig. 3).

**Table 1:** Sequences, Regions and product size of primers used in the analysis.

**Organism**	**Primers**	**Sequences**	**Region**	**Product size (bp)**
*Trypanosoma cruzi*	TC	5′CCCCCCTCCCAGGCCACACTG	Mini-Exon non transcribed spacer region for genotyping	
	TC1	5′GTGTCCGCCACCTCCTTCGGGCC		350 bp
	TC2	5′CCTGCAGGCACACGTGTGTGTG		300 bp

## 2 Experimental design, materials and methods

### 2.1 Pre-treatment and DNA extraction

Prior to DNA extraction, we macerated bone samples with a mortar and pestle in liquid nitrogen. Samples were re-suspended in a small amount of nuclease-free water for the sample to acquire adequate consistency for manipulation. The solution was incubated at 37 °C for 30 mi. This step was followed by DNA extraction using PureLink® Genomic DNA Kit (Invitrogen), according to the tissue sample protocol. We followed manufacturer׳s instructions, with the following modifications: samples were incubated with digestion buffers proteinase K and RNAse for 2hs and the DNA was eluted in a final volume of 50 µL, previously incubated at room temperature for 3 min and centrifuged for 2 minutes.

### 2.2 Amplification of products

We conducted a reconstructive PCR on the samples, as the DNA was fragmented and this is recommended to increase amplification product. This step does not add primers in the mix. Protocol was followed as described by Golenberg et al. [2] using conditions with the total volume of 25 µl: 1U Taq Platinum; 2.5 µl [10X] Buffer; 0.2 µl [25 mM] dNTP; 1.5 µl [2.5 mM] MgCl_2_. Cycling conditions were as followed: 94 °C 2 min; 20 cycles [94 °C 4 s; 50 °C 4 s; 72 °C 40 s].

After this step, PCR was conducted with amplification products of the reconstructive PCR and also with purified DNA. We conducted the protocol described by Fernandes et al. [3], with the following conditions: [1X] Buffer; [2.5 mM] MgCl_2_; [0.2 mM] dNTPs; 200 ng of each pair of primers; [2.5U] Taq; 5 µl DNA. Cycling conditions were as follows: 7′94 °C; 45 cycles [30″94 °C, 30″55 °C, 40″72 °C]; 7′72 °C. The pair of primers used were described by Souto et al. [4]. The authors amplified a part of the intergenic region of *Trypanosoma cruzi* mini-exon genes using a pool of oligonucleotides (see Table 1 for primers sequences and fragment sizes). These DNA markers define two phylogenetic lineages of *Trypanosonoma cruzi* (TC1 and TC2).

### 2.3 Sequencing and analysis

Amplified products were purified with Kit Wizard® SV Gel and PCR Clean-Up System (Promega), following manufacturers’ protocols. Obtained products were sequenced directly in both strips in an automatic sequencer (Applied Biosystems) by platform UFF/Instituto Biomédico. The softwares Chromas Lite 2.1 and BioEdit 7.2.5 were used to edit, analyze, and align sequences, which were compared to the Genbank database.

## Funding sources

This work was funded by FAPERJ (no. 202.785/2015), FUNDHAM, INAPAS, CAPES, Brazil.

## References

[1] Pucu E., Cascardo P., Chame M., Felice G., Guidon N., Vergne M.C., Campos G., Machado-Silva J.R., Leles D. (2017). Finding the unexpected: a critical view on molecular diagnosis of infectious diseases in archaeological samples. J. Arch. Sci. Rep..

[2] Golenberg E.M., Bickel A., Weihs P. (1996). Effect of highly fragmented DNA on PCR. Nucleic Acids Res..

[3] Fernandes A., Iñiguez A.M., Lima V.S., Mendonça de Souza S.M.F., Ferreira L.F., Vicente A.C.P., Jansen A.M. (2008). Pre-Columbian chagas disease in Brazil: *Trypanosoma**cruzi* I in the archaeological remains of a human in Peruaçu Valley, Minas Gerais, Brazil. Mem. Inst. Oswaldo Cruz.

[4] Souto R.P., Fernandes O., Macedo A.M., Campbell D.A., Zingales B. (1996). DNA markers define two major phylogenetic lineages of *Trypanosoma cruzi*. Mol. Biochem. Parasitol..

