# Supplementary material for: Study of infectious diseases in archaeological bone material – A dataset
Source: Data Brief. 2017 Jul 4;13:692–5. doi: 10.1016/j.dib.2017.06.054 (PMC5506867; doi:10.1016/j.dib.2017.06.054)
Supplement: Supplementary file 1 — Supplementary material [file mmc1.pdf]

I, Elisa Pucu, declare as first and correspondent author that there was no conflict of interest.

Elisa Pucu de Araujo
